# BatTS: a hybrid method for optimizing deep feedforward neural network

**DOI:** 10.7717/peerj-cs.1194

**Published:** 2023-01-10

**Authors:** Sichen Pan, Tarun Kumar Gupta, Khalid Raza

**Affiliations:** 1 School of Computer Science and Technology, Guangdong University of Technology, Guangzhou, Guangdong Province, China; 2 Department of Computer Science, Jamia Millia Islamia, New Delhi, Delhi, India

**Keywords:** ANN, Tabu search, Optimization

## Abstract

Deep feedforward neural networks (DFNNs) have attained remarkable success in almost every computational task. However, the selection of DFNN architecture is still based on handcraft or hit-and-trial methods. Therefore, an essential factor regarding DFNN is about designing its architecture. Unfortunately, creating architecture for DFNN is a very laborious and time-consuming task for performing state-of-art work. This article proposes a new hybrid methodology (BatTS) to optimize the DFNN architecture based on its performance. BatTS is a result of integrating the Bat algorithm, Tabu search (TS), and Gradient descent with a momentum backpropagation training algorithm (GDM). The main features of the BatTS are the following: a dynamic process of finding new architecture based on Bat, the skill to escape from local minima, and fast convergence in evaluating new architectures based on the Tabu search feature. The performance of BatTS is compared with the Tabu search based approach and random trials. The process goes through an empirical evaluation of four different benchmark datasets and shows that the proposed hybrid methodology has improved performance over existing techniques which are mainly random trials.

## Introduction

Since the mid of 1960s, different types of artificial neural network models (like feedforward neural network (FNN) and recurrent neural network (RNN)) have been suggested and tested for many real-world problems. However, the feedforward neural network is a prevalent model because of many reasons such as (a) it has structure flexibility, (b) it has a good representative capability, and (c) the existence of good training algorithms to train the FNN.

A simple feedforward neural network (also known as a multilayer perceptron) consists of an input layer, one hidden layer, and an output layer. The FNN has already been applied to many applications like signal processing (Hwang et al., 1997), classification and clustering (Zhang, 2000), speech processing (Gorin & Mammone, 1994), pattern recognition (Jain, Duin & Mao, 2000), function approximation (Selmic & Lewis, 2002), and cancer class prediction (Raza & Hasan, 2015).

The simple FNN is applicable only when the prediction function is not much complex or is not of very high dimensionality. Solving high dimensional nonlinear parts like classification in 100-classes or maybe more will not work with single hidden layers. FNN with more than one hidden layer is known as Deep feedforward neural network (DFNN). In this model, neurons (processing units) can be connected with next-level neurons only in the forward direction (*i.e*., no backward connection, no cycle, and no connection on the same level). In DFNN, the number of processing units at the input layer equals the number of input features. At the output layer, the number of processing units equals the number of classes in which data must be classified. This network can have various hidden layers and their respective neurons.

Selecting a proper design for DFNN is very important because it directly affects performance. DFNN with a small size can be stuck in underfitting, and if the size is considerable, the problem of overfitting arises. Most users perform hit-and-trial or random experiments to find a good DFNN model for a particular situation. However, these kinds of exercises will not guarantee an optimal architecture, and this kind of practice is a very time-consuming task. If anyone also considers the number of connections, it becomes very complex to adjust all these parameters.

When considering a simple FNN, which consists of single hidden layers, it needs to calculate the number of neurons only for that single hidden layer, while in the case of DFNN, it is pretty crucial. The uniqueness of this work is that it proposes a new approach which integrates the benefits of the Bat algorithm (Yang, 2010) and Tabu search (Glover, 1986) (BatTS). The aim of this work is to start with one hidden layer and optimize the single hidden layer FNN, then increase the hidden layer gradually and do the same task of optimization for multiple hidden layers. Finally, the BatTS used GDM (Li et al., 2009) to select an ideal model for DFNN. This whole scenario in earlier cases was accomplished as handcraft. The performance of the proposed methodology is then tested on four different datasets: (1) the MNIST dataset (LeCun & Cortes, 2010), (2) the ISOLET dataset (Dua & Graff, 2019), (3) the face recognition dataset (Ma, Correll & Wittenbrink, 2015) and (4) Gas Sensor Array Drift dataset (Rodriguez-Lujan et al., 2014; Vergara et al., 2012).

The article is structured into sections: “Related Work” briefly explains the literature review on optimizing the neural network using different methodologies. “Proposed Method” describes the proposed method and workings like solution representation, fitness function used, generation of population, and stopping criteria. “Datasets” discusses the used dataset with their properties. A detailed experimental setup with a summary of findings is presented in “Experimental Result”. Finally, “Conclusion” will provide conclusive remarks with some future scopes.

## Related work

Even though numerous studies have been done to develop a perfect ANN model, the automatic selection of a better ANN model for a specific objective is crucial. Over two decades, several strategies for optimizing training rules and ANN design have been presented. There can be many ways to automatically select the FNN model, such as constructing, pruning, model selection, nature-inspired techniques, hybrid techniques that combine two or more criteria, *etc*. Therefore, only a similar comparing algorithm is included in this section.

For example, the constructive methodology starts with a simple network and increases the network size iteratively according to the requirement. Mézard & Nadal (1989) proposed a new technique where hidden units can only be added when required and named this method a *tiling algorithm*. The authors guarantee that the architecture will converge for zero error. Frean (1990) presented different ways to construct FNN architecture and show that the newer architecture is less complex than Mézard & Nadal (1989). Zeng & Yeung (2006) defined the pruning technique to remove hidden neurons based on their relevance with the help of *quantified sensitive measures*. Islam et al. (2009) suggest a *new constructive methodology* for architecture adjustment. The main focus was to find hidden units for different layers. Augasta & Kathirvalavakumar (2011) predicted activation and weight functions. The proposed work was verified over benchmark datasets and showed its worthiness. Han & Qiao (2013) used constructive and pruning techniques in a hybrid manner for optimal FNN structure, but this was only applicable to FNN having only a single hidden layer.

To solve the issue of ANN architecture designing, a family of model selection approach like Akaike information criteria (AIC) (Akaike, 1974), root mean square (RMSE), mean absolute percentage error (MAPE), Bayesian information criteria (BIC) (Schwarz, 2007), direction accuracy (DA), *etc*., has been already studied. Murata, Yoshizawa & Amari (1994) proposed a novel model selection method for determining the appropriate hidden processing units. The methodology explores AIC on a shapeless model to find whether or not new units should be added to the network. Anders & Korn (1999) present a statistically acceptable model that starts with null architecture, and then hidden units were inserted one by one until the best architecture was identified. Aras & Kocakoç (2016) present new method *IHTS* (I = input, H = hidden, TS = trail selection). This new method helps determine input and hidden neurons using MSE, then validates the performance based on trial selection. Khaw, Lim & Lim (1995) showed how the Taguchi method can be implemented for neural network topology. Finally, Tortum et al. (2007) combine the Taguchi and model selection methods for optimizing FNN parameters.

On the other hand, many researchers use the evolutionary algorithm in ANN model prediction. Carvalho & Ludermir (2007) presented interleaved execution of two *Particle swarm intelligence* (PSO). In said work, the inner PSO optimizes weights, and the outer one optimizes topology. Yu, Xi & Wang (2007) explained the improved version of the PSO network (IPSONet) for architecture and weight process. Yu, Wang & Xi (2008), the authors presented the ESPNet result of the integration of PSO and DPSO (Kennedy & Eberhart, 1997) having flexible nature. PSO and DPSO dynamically adjust FNN topology, and self-addictiveness helps fine-tune the model. In Ludermir, Yamazaki & Zanchettin (2006), an integrated TS and SA approach was used to optimize a single-layer neural network. Gepperth & Roth (2006) optimized ANN architecture using a multi-evolutionary process.

In contrast, a novel approach GaTSa (Zanchettin, Ludermir & Almeida, 2011), integrates a genetic algorithm (GA), Tabu search (TS), and simulated annealing (SA) to optimize weight and architecture. In this method, GA worked constructively while TA and SA worked in a pruning way. In (Jaddi, Abdullah & Hamdan, 2015b), the authors present a new approach to finding optimal ANN topology and weights. Jaddi, in the same experiment, applied the Taguchi method to fine-tune the architecture. Later, the same experiment was performed with a multi-population-based cooperative bat algorithm (Jaddi, Abdullah & Hamdan, 2015a). Moreover, the authors (Jaddi, Abdullah & Hamdan, 2016) present a new dynamic model based on a genetic algorithm (GADNN). The representation contains two vectors; the first vector represents the hidden layers and individual neurons, while another represents weights according to the structure. The latest review in this domain can be seen in (Gupta & Raza, 2019). Gupta & Raza (2020) used Tabu Search and GDM to augment neural network architecture having more than one hidden layer. In this work, the author explores each layer exhaustively to search for optimal neurons for that particular layer. Finally, the algorithm decides how many layers and their respective neurons are necessary for a particular dataset. This experiment was performed over four benchmark datasets and shows that the proposed algorithm improves results. Later, the same experiment was performed using Bat Algorithm (Gupta & Raza, 2022). Pervaiz et al. (2021) shows a systematic analysis of PSO in health care. Bangyal et al. (2021) used a population-based algorithm for solving global optimization problems. Kuo, Kuruoglu & Chan (2022) developed a neural network design using simulated annealing rather than back-propagation for branch weight training.

In this literature recapitulation, we found a few critical flaws in FNN model optimization; they can be listed as (a) nearly all the algorithms work only for a single hidden layer neural network, with less dedication in the case of deep feedforward category, (b) merging and pruning techniques needs more devotion in pre-defining strategies, *i.e*., when and how to reject or accept hidden neurons in FNN and (c) In other algorithms the size of chromosomes is fixed which affect the performance; the manual description of chromosome dimension is problem specific.

The novelty of this article is to propose a hybrid method for optimizing hidden layers with their respective processing units, which can also work in the case of deep feedforward neural networks where hidden layers are more than one and the network could be more complex. This work will automatically increase or decrease the size of FNN architecture on the basis of probability (Algorithm 3).

## Proposed method

The aim of this section is to get introduce the Bat algorithm and Tabu search method. This section first includes the features of the Bat algorithm and explains the working of Bat with all of its equations and constants. Second, this section defines Tabu search with its properties and finally explains the working of our proposed method (BatTS).

### Bat algorithm

The bat algorithm (Yang, 2010) is a metaheuristic, swarm intelligence-based method. The algorithm is inspired by the echolocation behavior of microbats when it helps them find distances between themselves and their prey. In this algorithm, each bat has specific properties, *i.e*., frequency (*f*), velocity (*v*), position (*x*), loudness (*A*), and pulse rate (*r*). Initially, bats start randomly flying with a thunderous sound pulse and focus on the sound that bounced back from the object. The bounced-backed echo will tell about the distance, size, *etc*. The benefit of the bat algorithm is that it combines a swarm-based approach with a local search. The progressive iterations update each bat’s frequency, velocity, and position. In contrast, pulse and loudness adjusted only after accepting the new solution. The adjustment in frequency, velocity, and position can be made based on the following equations:

(1) $${f_i} = \; {f_{min}} + \left( {{f_{max}}\; - \; {f_{min}}} \right)\beta$$

(2) $$v_i^t = \; v_i^{t - 1} + \left( {x_i^{t - 1}\; - \; x_{gbest}^t } \right){f_i}$$

(3) $$x_i^t = \; x_i^{t - 1} + \; v_i^t$$

where $\beta \in \left[ {0,{\rm \; }1} \right]$ is a random number, ${f_i}$ represents the frequency of the *i*^th^ bat, velocity and positions of the *i*^th^ bat is denoted by ${v_i}$ and ${x_i}$ correspondingly. Also, $x_{gbest}^t$ is global best during iteration ‘t’.

Here a local search approach is applied to improve the variety of possible solutions based on certain conditions in the bat algorithm. Once the solution satisfies the condition, the random walk (Eq. (4)) strategy is used to populate a new solution:

(4) $${x_{new}} = {\rm \; }{x_{old}}{\rm \; } + {\rm \; \; }\varepsilon {A^t}$$

In Eq. (4), $\varepsilon$ represents a random number between the range of [−1, −1], and ‘*A*^*t*^*’* represents the mean loudness of the population in *t*^th^ iteration. As the bat moves toward its prey, typically, its loudness reduces, and its pulse rate rises. Loudness and pulse rate can be updated by using the following equations:

(5) $$A_i^{t + 1}\; = \; \propto A_i^t$$

(6) $$r_i^{t + 1}\; = \; r_i^0\left[ {1 - \exp \left( { - \gamma t} \right)} \right]$$

In Eqs. (5) and (6), $\propto$, and $\gamma$ are the constant where $\propto\ \in \left[ {0,{\rm \; }1} \right]$ and $\gamma > 0$.

### Tabu search algorithm

Tabu search is also a metaheuristic algorithm for finding the optimal global solution. The Tabu search method helps evaluate a group of solutions in each iteration, so it can reduce the cost of computation and can rapidly converge to an optimal solution. In Tabu search, if a solution is not improving, it can accept the lousy solution. This accepting bad solution property in Tabu search helps not get stuck in local minima/maxima. Tabu search algorithm, at each iteration, finds a local optimal *s’* and then compares this *s’* with *s*_*global*_. If *s’ < s*_*global*_, then *s*_*global*_ is updated by *s’*. To minimize repetition, the technique preserves a list (tabu list) of previously visited solutions.

### Optimization methodology

**Figure 1:**
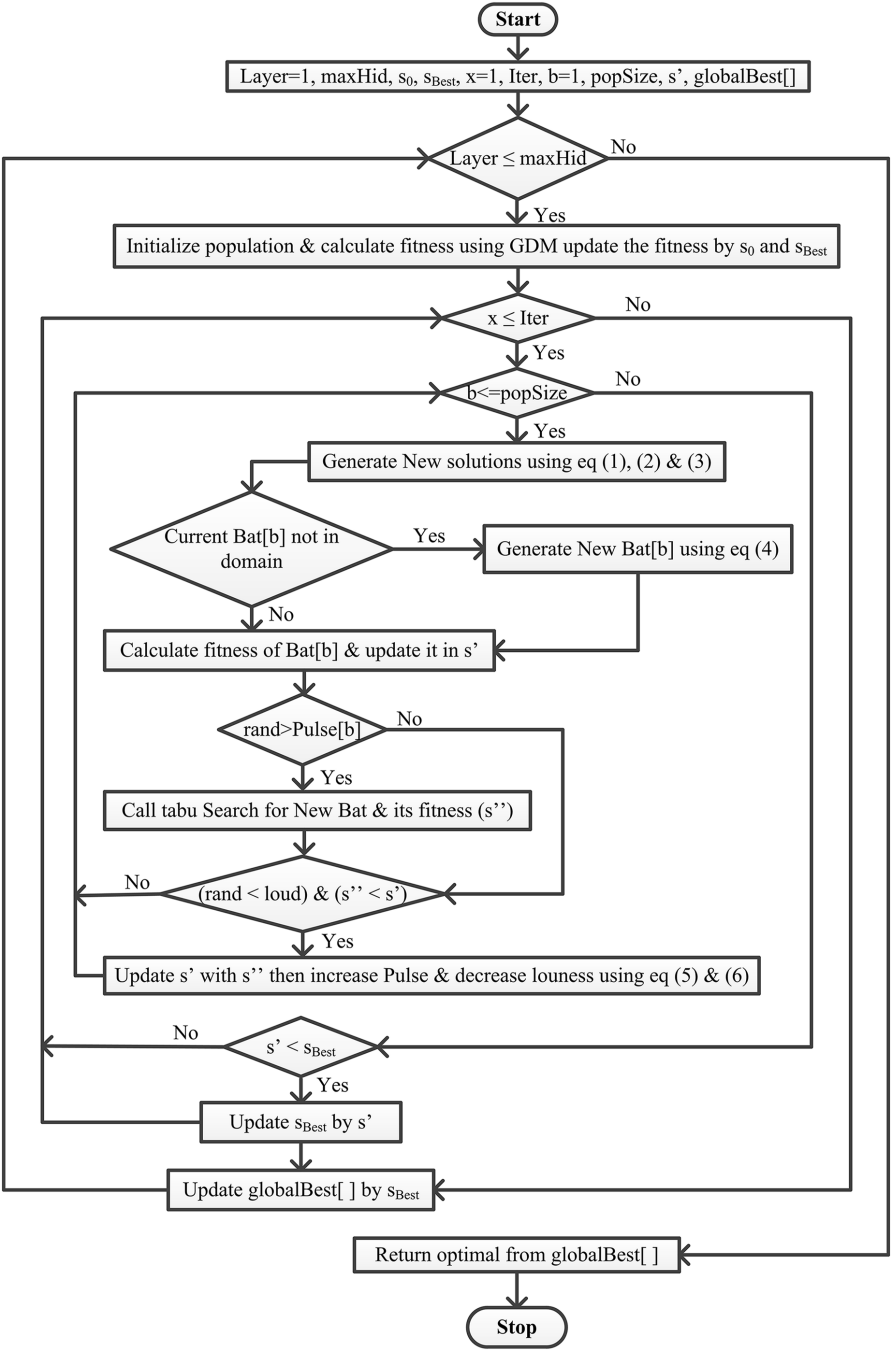
Flowchart of the proposed BatTS algorithm.

This article integrates the Bat algorithm with the Tabu search and GDM algorithm (Fig. 1). This work aims to find the best solution ${s_{Best}}$ from the given set of solutions *S*, where *f*( ${s_{Best}}$) ≤ *f*(*s’*), *f* is the objective function ∀*s’*∈ *S*. The proposed methodology starts with Algorithm 1 having a population of size ‘popSize.’ Then, the population in the said algorithm is initialized randomly, keeping in mind that ‘Bat[p]’ (actually the position of bat ‘p’) represents the neural network architecture. Bat[p] is a single-dimensional list, where the index of the list means hidden layer and the element represents respective processing units. Bat[p] initially has one hidden layer with randomly initialized processing units. The proposed algorithm works for maximum ‘Iter’ iterations and uses the tabu search to generate the local best solution rather than random generation. If the new best *f*(Bat[p]) is better than the current best *f*(s’) and has high loudness, then accept the new best, update this to the current best and increase pulse and decrease the loudness. Finally, update the current s’ best with ${s_{Best}}$ if *f*(*s’*) < *f*( ${s_{Best}}$).

**Algorithm 1:** Pseudocode for the proposed methodology.

**INPUT: ** ${\; }\# input\; neurons,\; \# output\; neurons,\; popSize,\; maxHid,\; Iter,\; minFreq,\; maxFreq$
${\; }\# input\; neurons,\; \# output\; neurons,\; popSize,\; maxHid,\; Iter,\; minFreq,\; maxFreq$
$minLoud,\; maxLoud,\; minPulse,\; maxPulse,\; minVel,\; maxVel,\; trainData,\; testData$
**OUTPUT**: $globalBest\left[ {} \right]$ //contains layer-wise best
${ for}\; Layer\; in\; maxHid\; :$
$I\; \leftarrow \; \# input\; neurons$
$O\; \leftarrow \; \# output\; neurons$
${ for\; \; }p\; in\; popSize:$ //Initialization of Population
$neuList\; \left[ {} \right]\; \leftarrow \; NULL$
$velList\; \left[ {} \right]\; \leftarrow \; NULL$
${ for}\; neurons\; in\; Layer:$ //calculate hidden neuron and velocity layer-wise
$a\; \leftarrow \; \left( {I + O} \right)/2$
$b\; \leftarrow \; \left( {I + O} \right)\times 2/3$
$neuList\; \left[ {neurons\; } \right]\; \leftarrow \; random\left( {a,b} \right)$
$velList\; \; \left[ {\; neurons} \right]\; \leftarrow \; random\left( {minFreq,\; maxFreq} \right)$
$I\; \; \leftarrow \; neuList\left[ {neurons} \right]$
$Bat\left[ p \right]\; \leftarrow \; neuList\left[ {} \right]$
$Vel[p]\leftarrow velist[]$
$Freq\left[ p \right]\; \leftarrow \; random\left( {minFreq,\; maxFreq} \right)$
$Pulse\left[ p \right]\; \leftarrow \; random\left( {minPulse,\; maxPulse} \right)$
$Loud\left[ p \right]\; \leftarrow \; random\left( {minLoud,\; maxLoud} \right)$
${s_0}\; \leftarrow \; calculateFitness\left( {Bat\left[ {popSize} \right],\; Layer} \right)$ //Using the GDM training Algorithm with a momentum of 0.7, ${s_0}$ is a structure of $Layer,\; neuList\left[ {} \right],\;$training error and testing error
${s_0}$ is the initial Solution update with ${s_{Best}}$
${ for\; }x\; in\; Iter:$
${ for\; }b\; in\; popSize:$
$generate\; new\; solution\; by\; adjusting\; Freq,\; Vel,\; Bat$
$using$ Eqs. (1)–(3)
$Control\; all\; new\; bats\; according\; to\; their\; domain\;$
${ if\; }Bat\left[ b \right]\; notin\; domain:$
$Bat\left[ b \right]\; \leftarrow \; update\; Bat\; by\; random\; walk$
${s^{\prime\; }}\; \leftarrow \; calculateFitness\left( {Bat\left[ b \right],\; Layer} \right)$
${ if\; }rand > Pulse\left[ b \right]:$
$Bat\left[ b \right],\; f\left( {Bat\left[ b \right]} \right)\; \leftarrow \; tabuSearch\left( {Bat\left[ b \right]} \right)$ //Calling Algorithm 2
//tabu search will return a new best Bat
${ if\; }rand < Loud\left[ b \right]\; and\; f\left( {Bat\left[ b \right]} \right) < f\left( {{s}^{\prime}} \right)\; :$
${s}^{\prime}\; \leftarrow f\left( {Bat\left[ b \right]} \right)$
$increase\; pulse\left[ b \right]\; and\; decrease\; Loud\left[ b \right]using$ Eqs. (5) and (6)
${ if}{s}^{\prime} \lt {s_{Best}}\; :$
$update\; {s_{Best}}\; \leftarrow \; {s}^{\prime}$
$globalBest\left[ {Layer} \right]\; \leftarrow \; {s_{Best}}$
$Return\left( {Optimal\left( {globalBest} \right)} \right)$

Algorithm 1 shows the proposed methodology with its pseudocode. For proper implementation, there is a particular condition that must be fulfilled first, like (a) representation of the solution, (b) fitness function, (c) generation of population, and (d) stopping conditions.

### Representation of solution

In this article, FNN with various hidden layers is taken for evaluation. All the connections are in the forward direction with no loop. Each neural network combines an Input layer with *‘I’* processing units, various hidden layers where *H*_*i*_ represents hidden units at the *i*^th^ layer, and an output layer with *‘O’* processing units. Basically, *‘I’* and *‘O’* are problem-dependent; the main task is to estimate the ideal number of hidden layers with their corresponding neurons. Mathematically, the structure of a neural network is represented as:

(7) $${N_{net}} \equiv \left( {I\; \times \; {H_1} + B\; \times \; {H_1}} \right) + \; \left( {{H_1} \times \; {H_2} + B\; \times \; {H_2}} \right) + \ldots + \; \left( {{H_{max}} \times \; O + B\; \times \; O} \right)$$

where B belongs to bias, in the proposed algorithm, every solution is represented by an array of four sections. The first section of the solution represents the number of hidden layers, *H*_L_, the second section of the solution represents a list of hidden neurons layer-wise, *H*_N_, the third section of the solution represents training error, *T*_tr,_ and the fourth section or the last section represents *T*_te_ testing error. Mathematically, the solution can be shown as:

(8) $$S\; \equiv \left( {{H_L},\; {H_N},\; {T_{tr}},\; {T_{te}}} \right)$$

(9) $${H_N}\; \equiv \left( {{H_1},\; {H_2},\; {H_3}, \ldots ,\; {H_{max}}} \right),\; \; \; \; \; {H_i}{\rm \; } \in {\rm \; {\mathbb N}}$$

${H_L}\; \equiv \left( {1,\; 2,\; 3,\; \ldots ,\; max} \right)$ and

(10) $${T_{tr}},\; {T_{te}}{\rm \; } \in {\rm \Re }$$

where ${\rm \Re }$ represents the real numbers set and ${\rm {\mathbb N}}$ represents the natural numbers set. In the representation, input and output units can be estimated by a given problem. In Contrast, at hidden levels, processing units are initialized by choosing randomly between the range of two thumb rules *i.e*., $\left[ {\displaystyle{{\left( {I + O} \right)} \over 2},\left( {I + O} \right) \times \displaystyle{2 \over 3}} \right]$. The weights are initialized using a uniform distribution [−1.0, +1.0].

### Fitness function

The accuracy of a populated model can be evaluated by using a fitness function. Recording the model’s fitness in all iterations is mandatory in any evaluation. Finally, it will return the model that optimizes that objective function. For example, considering the data is of *‘E*_*N*_*’* classes, let the true class of instance *‘d’* from training set *‘T’* can be defined as:

(11) $$\lambda \left( d \right) \in \left\{ {1,2,3,\; \ldots ,\; {E_N}} \right\}\; \; \; \forall d\; \in T$$

In this article, the proposed methodology implemented the “winner takes all” way, meaning the number of classes *C*_*N*,_ and neurons at output layers are equal.

If *O*_*P*_*(d)* is the output value of neuron *‘P’* at the output layer, for instance, *‘d’*, then the class for the given sample *‘d’* can be:

(12) $$\varphi \left( d \right) \equiv \arg ma{x_{p\; \in \left\{ {1,2,3,\; \ldots ,\; {C_N}} \right\}}}{O_P}\left( d \right)\; \; \; \; \; \forall d\; \in T$$

The error in the network, for instance, ‘*d*’ can be defined as follows:

(13) $$\varepsilon \left( d \right) \equiv \; \left\{ {\matrix{ {1,\; \; if\; \lambda \left( d \right) \ne \; \varphi \left( d \right)} \cr {0,\; \; if\; \lambda \left( d \right) = \; \varphi \left( d \right)\; } \cr } } \right.$$

Hence, for the training set *T*, the classification error *i.e*., incorrectly classified instances, can be calculated in percentage and represented as:

(14) $$E\left( T \right) \equiv \; \displaystyle{{100} \over {\# T}}\mathop \sum \limits_{d\; \in T} \varepsilon \left( d \right)$$

where *#T* is the number of instances in a given set *T*.

### Generation of population

In the proposed methodology, the generation of the population can occur in four different cases:

*Case 1*: This is the case when methodology starts for a particular hidden layer. Other population parameters are randomly initialized except for bat position (Bat [p]). At the same time, the bat position (represents network architecture) is initialized by choosing a number between the range of $\left[ {\displaystyle{{\left( {I + O} \right)} \over 2},\left( {I + O} \right) \times \displaystyle{2 \over 3}} \right]$. (*Bat [x, y] means solution having two hidden layers, and x, y is the respective neurons*).

*Case 2*: After getting the initial solution, the population of the bat is used to generate new solutions by using Eqs. (1)–(3).

*Case 3*: If the newly generated bat is not in the domain (the number of hidden neurons for any layer going outside the range), then use Eq. (4) to generate a new bat.

*Case 4*: When the *Pulse* of any bat is lesser, use tabu search to generate a new bat as shown in Algorithm 2.

**Algorithm 2:** Pseudocode for Tabu Search.

**INPUT: ** ${\; }Bat\left[ b \right],\; \; {s}^{\prime},\; Layer$ // Received from Algorithm 1
**INITIALIZE: ** ${\; }neuList\left[ {} \right]\; \leftarrow \; NULL$
$tabuTenure\; \leftarrow \; 4,\; {t_{best}}\; \leftarrow \; s^\prime$
$tabuList\; \leftarrow \; \left[ {\left[ {\; {t_{best}},\; tabuTenure} \right]} \right]$
${ for}\; x\; in\; range\; maxIter:$
$ {t}^{\prime}\; \; \leftarrow \; generateNeighbour\left( {Bat\left[ b \right]} \right):$ //calling Algorithm 3
$ {t}^{\prime}is\; the\; best\; with\; minimun\; testing\; error\; from\; neighbour\; of\; Bat\left[ b \right]$
$ l\; \leftarrow \; len\left( {tabuList} \right)$
$ Decrease\; tabuTenure\; of\; tabuList\; by\; one\; in\; last\; four\; solution$
$ {if}\; f\left( {{t}^{\prime}} \right) < f\left( {{t_{best}}} \right):$
$ tabuList\left[ {l + 1} \right]\; \leftarrow \; \left[ {\left[ {{t}^{\prime},\; \; tabuTenure} \right]} \right]$
$ {t_{best}}\; \leftarrow \; {t}^{\prime}$
$ Bat\left[ b \right]\; \leftarrow \; t^\prime$
$ {else}:$
$ {if}\left( {\left( {{t}^{\prime}\; ! = \; tabuList} \right)OR\left( {{t}^{\prime} = = tabuList\; AND\; tabuTenure = = 0} \right)} \right):$
$ tabuList\left[ {l + 1} \right]\; \leftarrow \; \left[ {\left[ {{t}^{\prime}\; ,\; \; tabuTenure} \right]} \right]$
$ Bat\left[ b \right]\; \leftarrow \; t^\prime$
$ {else}:$
$ Bat\left[ b \right]\; \; \leftarrow \; t^\prime$
$ Return\left( {{t_{best}}} \right)$// ${t_{best}}$ is the structure of $Layer,{\rm \; }neuList\left[ {} \right],$ training error and testing error

For better exploration, tabu search divides the list of generated neighbors (Algorithm 3) into two parts. The first part increases the hidden neurons by ‘*K*,’ and the second part decrease the hidden neurons by ‘*K*,’ see Eqs. (15) and (16):

**Algorithm 3:** Pseudocode for generating neighbors.

**INPUT:** *Bat, Layer* **// **Bat contains the number of neurons
**INITIALIZE: ** $\; {S_{max}}\; ,\; p\; ,\; K,\; candidateList\left[ {{S_{max}}} \right]\; \leftarrow \; NULL$
${ for}\; j\; in\; range\; {S_{max/2}}\; :$
$\hskip10pt g\; \leftarrow \; NULL$
$\hskip10pt { for}\; i\; in\; range\; Layer\; :$
$\hskip20pt r = random\left( {0,\; 1} \right)$
$\hskip25pt { if}\left( {r\; \ge p\; } \right)\; :$ // $p$ is a probability
$\hskip35pt Increase\; number\; of\; neurons\; by\; 'K'\; \; at\; that\; layer$
$\hskip18pt {else}\; :$
$\hskip35pt No\; change\; with\; neurons\; at\; that\; layer$
$\hskip20pt update\; neuList\; for\; candidateList\left[ j \right]$
$\hskip26pt g\; \leftarrow \; calculateFitness\left( {candidateList\left[ j \right]} \right)$ //Use GDM with Momentum 0.7
$\hskip18pt update\; g\; of\; candidate\left[ j \right]$
// ${\rm candidateList\; collection\; of\; Layer},{\rm \; neuList},{\rm \; testing},{\rm \; and\; training\; error}$
${ for}\; j\; in\; range\; {S_{max/2}}\; :$
$\hskip18pt g\; \leftarrow \; NULL$
$\hskip18pt { for}\; i\; in\; range\; Layer\; :$
$\hskip35pt r = random\left( {0,\; 1} \right)$
$\hskip35pt { if\; }\left( {r\; \le \; p\; } \right)\; :$
$\hskip45pt Decrease\; number\; of\; neurons\; by\; ^\prime K^\prime\; \; at\; that\; layer$
$\hskip35pt {else}\; :$
$\hskip45pt No\; change\; with\; neurons\; at\; that\; layer$
$\hskip18pt update\; neuList\; for\; candidateList\left[ {j + \; {S_{max/2}}} \right]$
$\hskip25pt g\; \leftarrow \; calculateFitness\left( {candidateList\left[ j \right] + \; \; {S_{max/2}}} \right)$ //Use GDM with Momentum 0.7
$\hskip15pt update\; g\; of\; candidate\left[ j \right]$
$Return\; best\; of\; candidateList$

(15) $${H_N} = \; \left\{ {\matrix{ \;\;\;\;K + ,  \;\;\;\;\;\;\;\;\;\;r\; \ge p \cr no\; change,  \;\;\;r\; \lt p \cr } } \right.$$

and,

(16) $${H_N} = \; \left\{ {\matrix{ \;\;\;\;\;K - ,  \;\;\;\;\;\;\;r\; \ge p \cr no\; change,  \;\;r\; \lt p \cr } } \right.$$

Here, *‘r’* is the uniformly distributed [0, 1] random number, and *‘p’* is the probability of change in neurons.

### Stopping criteria

In this work, the optimizations stop if it reaches maximum iteration. Furthermore, there are some other conditions also when the algorithm will stop updating the hidden neurons and switch to successive steps: (i) when it tries to add hidden neurons using Eq. (15) while the network reaches its maximum limit, (ii) when it tries to subtract hidden neurons using Eq. (16) while the network reaches to its lower limit.

## Datasets

This section describes the datasets used in this article to validate the proposed methodology. This proposed methodology aims to optimize DFNN *i.e*., a neural network with multiple hidden layers, so it is mandatory to opt for a dataset with an extensive range of input features that can be classified into multiple classes. Otherwise, with a small feature dataset, the proposed algorithm will not be validated efficiently and will converge to a simple neural network. We have used four different benchmark datasets comprised of different numbers of examples, features, and classes. A summary of the used datasets is shown in Table 1.

**Table 1:** Datasets statistics considered for the experiments.

Dataset	Examples	Features	Classes	References
Face	1,846	784	2	Ma, Correll & Wittenbrink (2015)
Gas-drift	13,910	128	6	Vergara et al. (2012), Rodriguez-Lujan et al. (2014)
MNIST	70,000	784	10	LeCun & Cortes (2010)
ISOLET	7,797	617	26	Dua & Graff (2019)

### Experimental result

As discussed in previous sections, choosing the correct architecture for a neural network is crucial and time-consuming. To estimate the configuration, hit and trial experiments are in trend, but this will not guarantee ideal architecture. Therefore, this section includes the findings in random experiments and BatTS experiments.

All experiments are implemented in R using the H_2_O package. The implemented methodology uses multinomial distribution, 0.2 dropout ratio, 0.7 momentum term, and 0.0001 learning rate. Each model is validated by using 20% of each dataset as a validation dataset.

### Random experiments

Generally, in random trial-based experiments, the user repeatedly selects a neural network model by changing its parameters manually and running these models on a given dataset. Finally, the best one based on minimum testing/training error is chosen. Here is a random experiment for every dataset, we run 30 different topologies for a single hidden layer, then 30 different topologies for two hidden layers, and so on up to *maxHid* = 5. We fixed the maximum hidden layer (*maxHid*) = 5 which can be increased on the basis of problem complexity. Neurons for hidden layers were selected randomly by using a thumb rule [(I+O)/2, (I+O) × 2/3]. Where *‘I’* is the Input features and *‘O’* is the output classes. At the end, we choose the best architecture on the basis of minimum testing error. Every network selected for evaluation was fully connected and trained by using GDM. The results are shown in Table 2.

**Table 2:** Experimental statistics in terms of mean square error (MSE). Bold shows optimal architecture.

Data set	H_L_	Proposed methodology			Tabu search based algorithm (Gupta & Raza, 2020)			Random experiments (Gupta & Raza, 2020)		
		Hidden Neurons at each layer	MSE (%)		Hidden Neurons at each layer	MSE (%)		Hidden Neurons at each layer	MSE (%)	
			Train	Test		Train	Test		Train	Test
Face	1	508	2.783	10.605	515	2.612	11.396	476	1.022	13.976
	2	**423, 259**	7.429	**9.281**	**437, 260**	8.0481	**10.385**	**499, 262**	5.93	**12.561**
	3	512, 273, 138	9.352	10.785	506, 261, 140	9.568	11.438	398, 217, 134	10.14	13.387
	4	460, 248, 136, 88	8.021	10.916	454, 261, 148, 73	8.491	11.007	497, 254, 168, 100	9.65	14.17
	5	508, 267, 157, 95, 58	11.073	12.253	445, 250, 156, 189, 48	11.651	13.176	490, 245, 136, 80, 53	11.799	14.212
Gas-Drift	1	85	5.301	6.041	75	5.317	6.347	82	5.611	6.875
	2	**83, 56**	3.836	**4.208**	**90, 62**	4.986	**5.024**	**83, 52**	5.061	**6.374**
	3	75, 48, 30	6.118	5.632	78, 53, 36	6.046	6.295	81, 52, 33	5.559	7.006
	4	81, 52, 36, 22	10.4077	9.58	74, 49, 35, 25	10.5086	10.707	75, 48, 32, 21	12.0165	11.922
	5	78, 47, 32, 20, 14	25.856	23.472	79, 45, 33, 25, 19	25.455	25.784	86, 54, 35, 24, 17	36.5256	36.212
MNIST	1	**522**	0.628	**1.6501**	**518**	0.651	**1.823**	**521**	0.761	**1.893**
	2	528, 278	0.742	1.898	547, 225	0.645	1.902	416, 228	0.937	1.921
	3	512, 328, 210	1.206	1.907	520, 289, 165	1.111	1.926	415, 214, 126	1.031	2.016
	4	494, 298, 175, 95	1.264	2.001	532, 302, 130, 75	1.289	2.057	442, 262, 141, 81	1.308	2.008
	5	448, 278, 173, 110, 61	4.438	3.131	490, 274, 158, 103, 64	3.232	3.081	406, 258, 150, 84, 53	8.095	7.843
ISOLET	1	348	0.073	2.18	335	0.0301	2.069	357	0.022	2.289
	2	**355, 198**	0.247	**1.532**	**362, 231**	0.272	**1.798**	**403, 211**	0.255	**2.664**
	3	414, 261, 155	1.994	2.186	397, 212, 119	0.851	2.393	322, 170, 103	1.199	2.635
	4	393, 234, 126, 79	3.365	4.135	406, 254, 155, 100	3.312	4.655	356, 204, 137, 72	10.4142	11.63
	5	424, 263, 157, 94, 63	92.276	91.334	402, 227, 135, 78, 46	92.448	91.484	381, 240, 132, 78, 48	92.452	92.4643

For the face classification, the optimal architecture returns by random experiments having two hidden layers with {499, 262} hidden neurons. The topology gives a 12.561% mean square error (MSE). The best architecture for the gas drift dataset received two hidden layers holding {83, 52} units and MSE as 6.473%. In the case of the MNIST data, the MSE of 1.839% was calculated with single hidden layer architecture and 521 hidden units. The best performance for the ISOLET dataset measured MSE as 2.266%. The topology for ISOLET using random experiments received with two hidden layers having {403, 211} neurons for respective hidden layers.

### BatTS experiments

The BatTS starts with one hidden layer network and randomly chosen hidden units. After creating this as the initial topology, the fitness function is calculated with the help of the H_2_O package and recorded as the best solution. Every solution here in this BatTS is iterated by *Iter* = 10, and then in every iteration, every solution will create a population of size, *popSize* = 20. If the generated solution is not in the domain (*i.e*., hidden units are going outside the lower and upper range), then a random walk is used to create the new solution and adjust this in the domain for any bat having a *Pulse* less than a certain threshold, Tabu search (Algorithm 2) help to generate a new solution and return its fitness value. A bat is only accepted if fitness is optimal and loudness is high. Once the solution is accepted, the *Pulse* is increased, and Loudness, *Loud* is decreased. The BatTS runs for *maxHid* = 5. The probability of changing neurons that is, one can increase or decrease (in Algorithm 3), *p* = 0.5. The size of the architecture is increased or decreased by K% = 3.

The experimental statistics are presented in Table 2. In the case of the face dataset, BatTS proposes architecture with *H*_*L*_= 2 and the respective neurons are {423,259}. The mean square error (MSE in percentage) was 9.281%. For Gas-Drift, the optimal architecture suggested by the proposed algorithm was H_L_ = 2 with {83,56} hidden processing units, and the MSE was 4.208%. In the case of MNIST, MSE return by the algorithm was 1.6501, while the optimal topology has H_L_= 1 with {522} hidden units. For the ISOLET, the optimal configuration required Hidden layers, H_L_ = 2 and H_N_ = {355, 198}, and the MSE noted as 1.532%.

**Figure 2:**
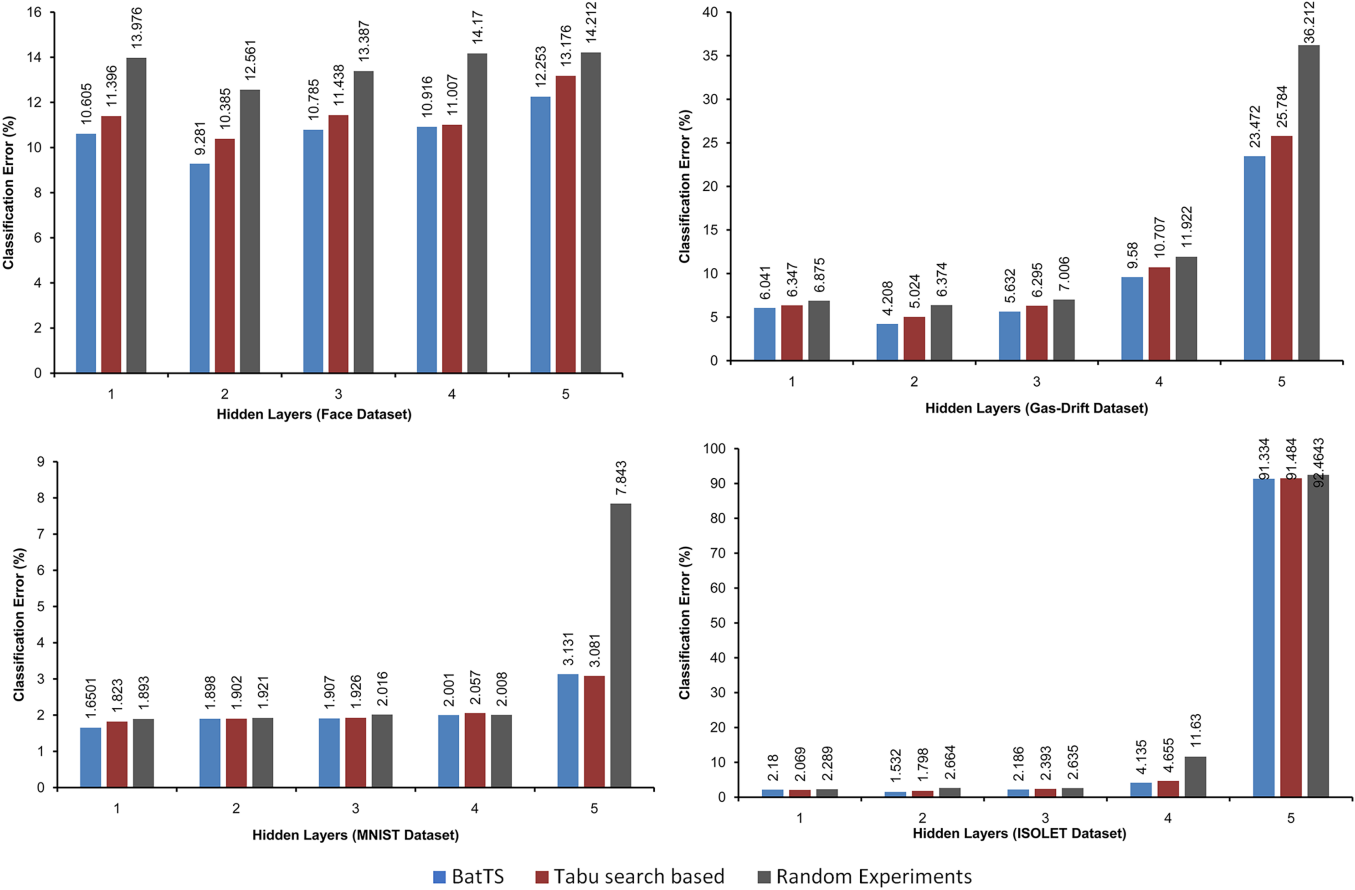
Performance of the BatTS and its comparison with Tabu search based methodology and random experiments.

**Figure 3:**
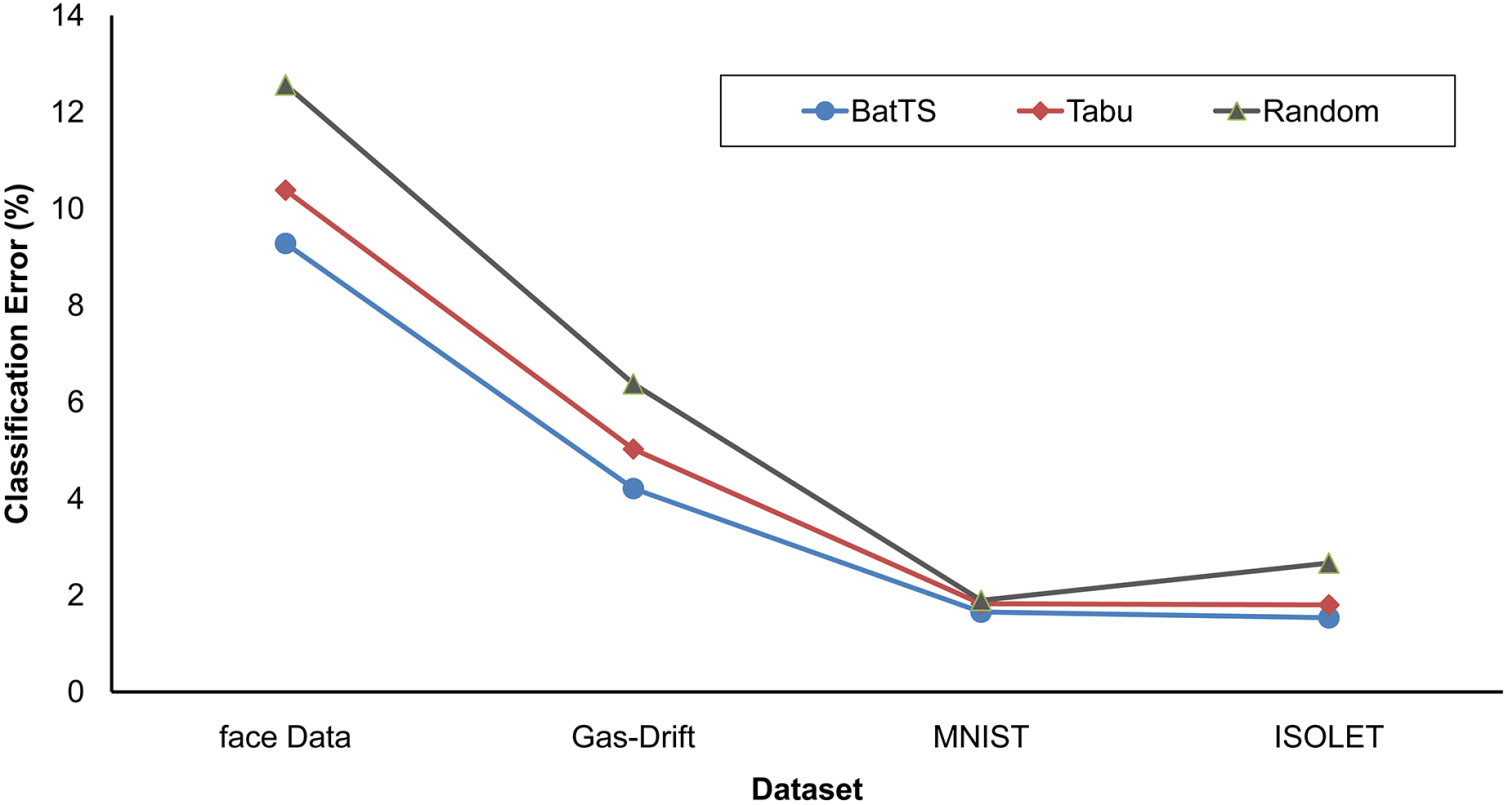
Comparison of BatTS with Tabu search based methodology and random experiments.

The fitness function is evaluated based on testing error rather than training error. Table 2 shows that BatTS performed better than Random experiments and the Tabu-based approach (Gupta & Raza, 2020). Figs. 2 and 3 present the performance comparison of the proposed BatTS method with the Tabu search based method and random experiments on the four benchmark datasets which clearly shows a significant reduction in the classification error.

## Conclusion

This work aims to estimate the ideal number of hidden layers with respective hidden units for deep feedforward neural networks. We consider minimum testing mean square error for the optimal network. Another important task was to take a dataset with significant input features that must be classified into large classes. If the input size of the dataset is less than no meaning of deep feedforward neural network, it may converge to a simple network. The methodology shows that if the bat is integrated with TS, it can generate optimal topology, which can be hard to predict in hit and trial experiments. Table 2 shows that BatTS finds a better network than the tabu-based approach.

The proposed methodology also shows that there is no requirement for predefining the way of merging and pruning. This strategy does not fix the size of the solution which can affect the performance of the methodology. The network considered here is fully connected with more than one hidden layer; the algorithm can be improved if applied to different convolutional neural networks (CNN) like VGG, DenseNet, or ResNet. In these variants, the FNN portion is fixed, and the BatTS may also show some better findings. Moreover, many other natures inspired algorithms like PSO, Ant Colony Optimization, *etc*., may help develop this kind of methodology for optimal DFNN architecture.

## Additional Information and Declarations

### Data Availability

The following information was supplied regarding data availability: The code and the data are available at Zenodo: Tarun Kumar Gupta. (2022). BatTS [Data set]. Zenodo. https://doi.org/10.5281/zenodo.7321656.
